# The signature of cuproptosis-related immune genes predicts the tumor microenvironment and prognosis of prostate adenocarcinoma

**DOI:** 10.3389/fimmu.2023.1181370

**Published:** 2023-08-02

**Authors:** Kai Yao, Rumeng Zhang, Liang Li, Mingdong Liu, Shiyao Feng, Haixin Yan, Zhihui Zhang, Dongdong Xie

**Affiliations:** ^1^ Department of Urology, The Second Affiliated Hospital of Anhui Medical University, Hefei, China; ^2^ Department of Pathology, School of Basic Medicine, Anhui Medical University, Hefei, Anhui, China; ^3^ Department of Urology, Affiliated Fuyang Hospital of Anhui Medical University, Fuyang, Anhui, China

**Keywords:** cuproptosis, PrlR, des, LECT2, prostate cancer

## Abstract

**Background:**

Cuproptosis plays a crucial role in cancer, and different subtypes of cuproptosis have different immune profiles in prostate adenocarcinoma (PRAD). This study aimed to investigate immune genes associated with cuproptosis and develop a risk model to predict prognostic characteristics and chemotherapy/immunotherapy responses of patients with PRAD.

**Methods:**

The CIBERSORT algorithm was used to evaluate the immune and stromal scores of patients with PRAD in The Cancer Genome Atlas (TCGA) cohort. Validation of differentially expressed genes DLAT and DLD in benign and malignant tissues by immunohistochemistry, and the immune-related genes of DLAT and DLD were further screened. Univariable Cox regression were performed to select key genes. Least absolute shrinkage and selection operator (LASSO)–Cox regression analyse was used to develop a risk model based on the selected genes. The model was validated in the TCGA, Memorial Sloan-Kettering Cancer Center (MSKCC) and Gene Expression Omnibus (GEO) datasets, as well as in this study unit cohort. The genes were examined *via* functional enrichment analysis, and the tumor immune features, tumor mutation features and copy number variations (CNVs) of patients with different risk scores were analysed. The response of patients to multiple chemotherapeutic/targeted drugs was assessed using the pRRophetic algorithm, and immunotherapy was inferred by the Tumor Immune Dysfunction and Exclusion (TIDE) and immunophenoscore (IPS).

**Results:**

Cuproptosis-related immune risk scores (CRIRSs) were developed based on PRLR, DES and LECT2. High CRIRSs indicated poor overall survival (OS), disease-free survival (DFS) in the TCGA-PRAD, MSKCC and GEO datasets and higher T stage and Gleason scores in TCGA-PRAD. Similarly, in the sample collected by the study unit, patients with high CRIRS had higher T-stage and Gleason scores. Additionally, higher CRIRSs were negatively correlated with the abundance of activated B cells, activated CD8^+^ T cells and other stromal or immune cells. The expression of some immune checkpoints was negatively correlated with CRIRSs. Tumor mutational burden (TMB), mutant-allele tumor heterogeneity (MATH) and copy number variation (CNV) scores were all higher in the high-CRIRS group. Multiple chemotherapeutic/targeted drugs and immunotherapy had better responsiveness in the low-CRIRS group.

**Conclusion:**

Overall, lower CRIRS indicated better response to treatment strategies and better prognostic outcomes.

## 1 Introduction

Prostate adenocarcinoma (PRAD) is a major disease affecting the health of men worldwide and is the second most common malignancy among men (1). In 2020, more than 1.4 million new cases of PRAD were reported worldwide (2). Recent changes in acquired risk factors have led to an increase in the incidence of PRAD in Asian countries (3). Radical prostatectomy (RP) or radiotherapy is the standard treatment for most patients with local PRAD (4). However, biochemical relapse occurs in 30%–50% of patients after treatment (5). Approximately 20% of intermediate-risk patients experience biochemical failure within 18 months of initial local treatment (6, 7). The oncogenic mechanisms underlying PRAD remain unclear, and targeted therapy, especially for high-risk PRAD and castration-resistant prostate cancer (CRPC), remains challenging (8, 9). Therefore, an in-depth understanding of the multiple characteristics of PRAD and the identification of effective prognostic indicators can help to develop more effective treatment strategies for PRAD.

Copper is an indispensable trace element involved in biological processes in eukaryotes, including iron transport, oxygen free radical detoxification and mitochondrial respiration (10). The intracellular copper concentration is in a dynamic gradient-based equilibrium and various cellular processes such as lipolysis, proliferation and autophagy are regulated by this dynamic signal (11–15). Owing to the dysregulation of copper transmembrane transport, intracellular copper accumulation leads to cytotoxicity and cell death (16). Excess copper increases intracellular reactive oxygen species (ROS) levels, induces endoplasmic reticulum stress, enhances damage-related molecular patterns and promotes macrophage phagocytosis (17). Peter et al. identified a novel mechanism by which copper induces cell death: copper directly binds to the lipoacylated components of the tricarboxylic acid (TCA) cycle, leading to toxic protein stress and, eventually, cell death (18). They also identified seven genes positively associated with cupviaroptosis, including FDX1, LIAS, LIPT1, DLD, DLAT, PDHA1 and PDHB. Cuproptosis is a new cell death mechanism that is different from necrosis (19), apoptosis (20), necroptosis (21), autophagy (22), pyroptosis (23), oxeiptosis (24), parthanatos (25) and ferroptosis (26). Copper importers (SCL31A1) and exporters (ATP7A and ATP7B) are key genes that regulate and maintain intracellular copper concentration (18). Mutations in the ATP7A and ATP7B genes can lead to deficiency and accumulation of copper, leading to Menkes and Wilson diseases, respectively. Supplementation or removal of copper represents a novel therapeutic strategy for neurodegenerative diseases (27).

Copper may also play a role in the pathogenesis and progression of cancer (28, 29). Elevated serum copper levels are associated with tumor stage and disease progression in patients with colorectal, lung and breast cancers (30–32). Daily administration of copper sulfate (CuSO4) has been shown to increase tumor growth in a rat model of chemically induced mammary tumors (33). The cuproenzyme LOX is involved in the invasion and metastasis of tumor cells (34). In a mouse model of breast cancer, knockdown of ATP7A reduced LOX activity, decreased the recruitment of bone marrow cells to the lung, and inhibited tumor growth and metastasis (35). Further, it has been reported that patients with high expression of FDX1, SDHB, DLAT and DLST in colorectal cancer tissues have a better prognosis (36). In hepatocellular carcinoma, characteristics based on cuproptosis patterns are important for predicting the tumor microenvironment (TME) and immunotherapy responses (37). Cuproptosis features can also help to predict the prognosis and immune microenvironment of patients with breast cancer (38). Copper chelators can be used as antiangiogenic agents to alter the TME (39) and enhance antitumor immunity (40) in various cancers (39). However, the role of cuproptosis in prostate adenocarcinoma (PRAD) remains unclear. An in-depth study on the impact of cuproptosis on the immune landscape of PRAD may help to elucidate the role of cuproptosis in PRAD and identify novel therapeutic targets.

In this study, we clustered and analysed alterations in immune-related genes associated with two subtypes of cuproptosis with different prognostic features. We developed a new metric named ‘cuproptosis-related immune risk score’ (CRIRS) based on cuproptosis- and immune-related genes to assess the immune characteristics and prognosis of patients with PRAD. Additionally, immune-related components, metabolic characteristics, and gene mutation profiles were analysed in different risk groups, and the results showed significant differences in these aspects between the high- and low-risk groups. The predictive staging model showed great potential to guide the classification of patients with PRAD and predict the chemotherapy and immunotherapy responses of risk-stratified patients. Overall, the model exhibited potential clinical value.

## 2 Materials and methods

### 2.1 Data collection

Survival data, clinical information and mRNA expression data, CNV and somatic mutation data for PRAD in the TCGA dataset downloaded from the UCSC-Xena database (https://xenabrowser.net/datapages/). The Memorial Sloan Ketterring Cancer Center (MSKCC)-PRAD database (Cancer Cell 2010, https://www.cbioportal.org/) and Gene Expression Omnibus (GEO) database (GSE70770, https://www.ncbi.nlm.nih.gov/geo/query/acc.cgi?acc=GSE70770) were used as validation sets (**Supplementary Table S1**). Samples without important clinical or survival data were excluded from further analysis. Immune-related genes were extracted from ImmPort Shared Data (http://www.immport.org). Raw reads were post-processed and normalized using the ‘DESeq2’ (version 1.38.3) package in the R (version 4.2.0) software.

### 2.2 Estimation of stromal and immune cells

The CIBERSORT algorithm was used to assess the proportion of immune cell subpopulations in each PRAD sample (41). The single-sample gene set enrichment analysis (ssGSEA) algorithm was used to assess the levels of human leukocyte antigens (HLAs), immune cell infiltration and immune cell function (42). In addition, the proportion of 64 cell types in the TME of patients in TCGA-PRAD cohort was assessed using the xCell algorithm, and elements of TME, including immune, stromal and microenvironment scores, were estimated (43).

### 2.3 Consensus clustering

To examine the effects of cuproptosis on the immune function of patients with PRAD, the correlation between the expression of cuproptosis-related positive regulators and CIBERSORT results was examined *via* Spearman analysis. The R package ‘ConsensusClusterPlus’ was used for consensus clustering of tumor samples based on the expression of DLAT and DLD and for visualisation of the results (44). The Kaplan–Meier method and log-rank test were used to compare OS between two clusters.

### 2.4 Analysis and validation of scRNA data

IMMUcan Database (https://immucanscdb.vital-it.ch/) is a comprehensive tumor microenvironment database platform that mines the single cell characteristics of tumor immune microenvironment based on a large collection and integrated analysis of single cell data (45). To validate the expression of DLAT and DLD in prostate cancer immune cells, the prostate cancer single-cell sequencing dataset GSE141445 was analyzed using the UMAP algorithm in the IMMUcan Database.

### 2.5 Differentially expressed genes and cuproptosis-related immune scores

**Figure 1:**
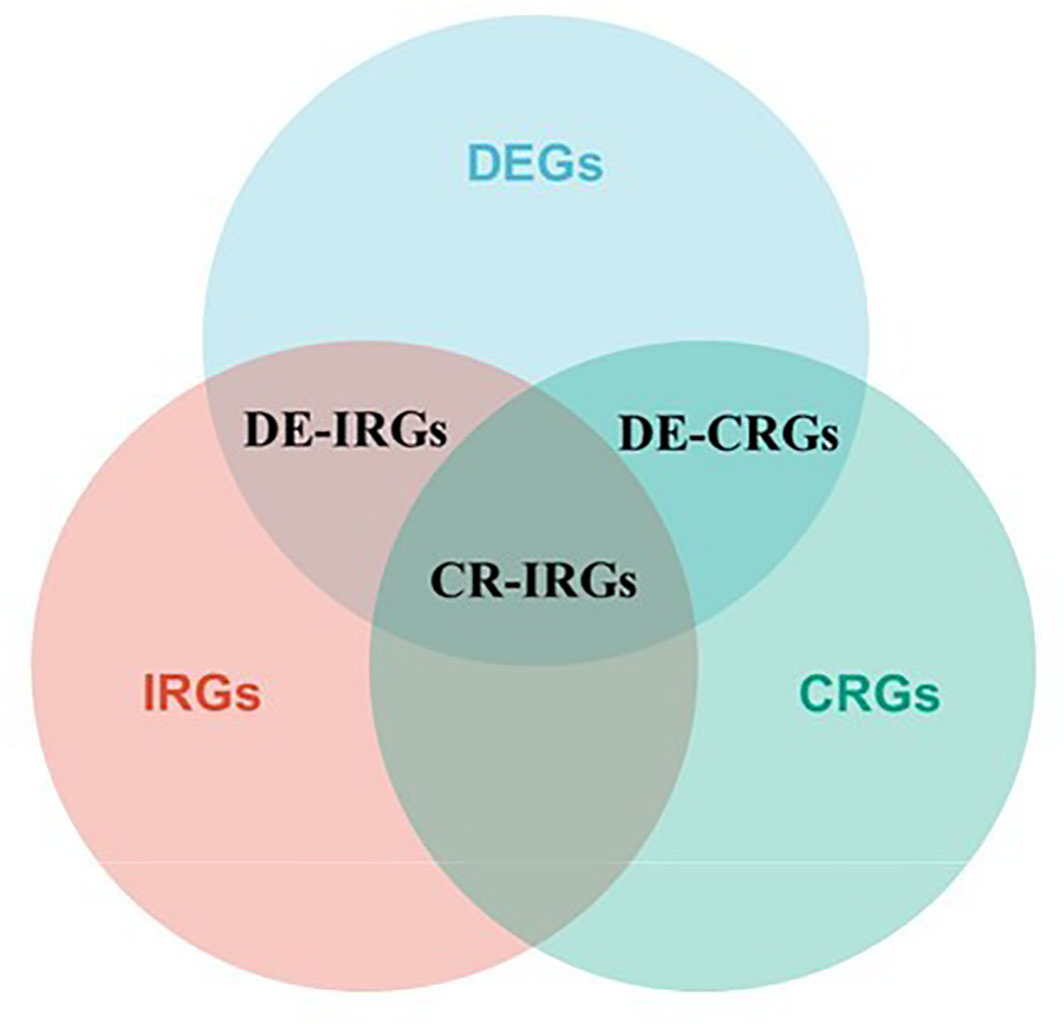
Venn diagram of the CR-IRGs screening process.

Differentially expressed genes (DEGs) in cancerous and paraneoplastic tissues were identified using the ‘DESeq2’ package in R in TCGA-PRAD cohort, with the threshold set as log2 foldchange (FC) values of ≥1 and FDR < 0.05. Pearson correlation analysis was performed to select DEGs associated with DLAT and DLD (cor > 0.3, *P* < 0.05), named cuproptosis-related DEGs (CR-DEGs). On the other hand, crossover between immune-related genes and DEGs was performed to obtain immune-related DEGs (IR-DEGs); the latter immune-related genes (n = 2,483) were extracted from the Immunology Database and Analysis Portal (ImmPort, https://www.immport.org/) database. The cuproptosis- and immune-related genes are the intersecting genes of CR-DEGs and IR-DEGs (CR-IRGs). The screening process of CR-IRGs is shown in **Figure 1**. The potential function of these CR-DEGs and CR-IRGs was then determined by Gene Ontology (GO) annotation and Kyoto Encyclopedia of Genes and Genomes (KEGG) enrichment pathway analysis using the “clusterProfiler” package in R. Univariable Cox regression analysis was performed to screen for CR-IRGs related to the prognosis of PRAD (*P* < 0.05). Subsequently, a CR-IRGs signature was constructed *via* least absolute shrinkage and selection operator (LASSO)–Cox regression analysis. The risk score was calculated as follows: Risk score = ∑Coefi∗Expi , where Coefi represents the coefficients and Expi represents the expression levels of the three key genes.

### 2.6 Functional enrichment analysis

The ‘GSVA’ (version 1.30.0) package was used to identify the different pathways associated with cuproptosis-related genes and analyse the relationship between CRIRSs and HALLMARK pathways. Heatmaps were drawn using the ‘heatmap’ package in R to visualise the results. GSEA was performed for CRIRS-based classification of patients with PRAD. Line plots were drawn using the ‘ggplot2’ package in R.

### 2.7 Survival analysis

FPKM method was used to normalize the raw data. Based on the best survival cut-off grouping, we classified patients into high- or low-CRIRS groups. For Kaplan–Meier curves, *P*-values and hazard ratios (HRs) with 95% confidence intervals (CIs) were calculated *via* the log-rank test. HRs of > 1 indicated risk factors, whereas HRs of < 1 indicated protective factors. The R packages ‘survival’, ‘survminer’ and ‘timeROC’ were used for survival analysis. *P*-values of < 0.05 were considered statistically significant. More importantly, 1-year, 3-year and 5-year prognostic values for OS and DFS, survival-dependent subject operating characteristic (ROC) curves and calibration curves were used to evaluate the CRIRS model in the TCGA training set and robustly validated in the MSKCC and GSE70770 cohorts.

### 2.8 Correlations between CRIRS model and clinical characteristics

A subgroup analysis of the three signature genes in the prognostic profile associated with cuproptosis was performed according to the clinical characteristics of the patients. Next, univariable and multivariable Cox regression analyses were performed to determine the prognostic role of the CRIRS model. The ‘forestplot’ R package was used to draw a forest plot to demonstrate *P*-values, HRs and 95% CIs for each variable. Then, the association between CRIRSs and each clinical parameter was further analyzed and presented by boxplots and pieTable.

### 2.9 Quantitative real-time PCR

Total RNA was extracted from paraffin-embedded tissues using a reliable RNA-isolation kit from Thermo Fisher Scientific, USA. The mRNA levels of specific genes, PRLR, DES and LECT2, were measured by qRT-PCR using SYBR green Master MIX from Applied Biosystems, which fluoresces when it binds to double-stranded DNA during the PCR reaction. GAPDH was used as an endogenous control. The primer sequences are presented below: GAPDH: 5’- TGGCCATTATAGGACCGAGACTT -3’ (forward) and 5’- CACCCTGTTGCTGTAGCCAAA -3’ (reverse); PRLR: 5’- TCTCCACCTACCCTGATTGAC -3’ (forward) and 5’- CGAACCTGGACAAGGTATTTCTG -3’ (reverse); DES: 5’- TCGGCTCTAAGGGCTCCTC -3’ (forward) and 5’- CGTGGTCAGAAACTCCTGGTT -3’ (reverse); LECT2: 5’- TGGGCCAGGAGAAACCTTATC -3’ (forward) and 5’- CAAGGGCAATAGAGTTCCAAGTT -3’ (reverse).

### 2.10 Immunohistochemistry

Immunohistochemistry (IHC) was utilized to evaluate the protein expression of DLD and DLAT in paraffin sections obtained from patients diagnosed with prostate cancer and benign prostatic hyperplasia. Mouse monoclonal antibodies (Proteintech Group, Inc, Chicago, USA) for DLAT (1: 1000) and DLD (1:500) were used, respectively. All tissue information on the sections was captured using the Panoramic MIDI (manufacturer: 3D HISTECH).

### 2.11 Frequency of somatic mutations and copy number variations

The somatic mutation data of TCGA-PRAD cohort were extracted in the varscan file format. CNV data were downloaded from UCSC Xena (https://xenabrowser.net/datapages/). To determine the somatic mutation patterns of patients with PRAD in the high- and low-CRIRS groups, the data were converted into the mutation annotation format (MAF) using the ‘maftools’ R package. Tumor mutation burden (TMB) and mutant-allele tumor heterogeneity (MATH) scores were also evaluated in both groups.

### 2.12 Chemotherapy and immunotherapy drug sensitivity

The Genomics of Drug Sensitivity in Cancer (GDSC; https://www.cancerrxgene.org/) database was used to assess the sensitivity of each patient to several chemotherapeutic agents, and the half-maximal inhibitory concentration (IC50) was quantified using the ‘pRRophetic’ package in R. The response to immune checkpoint blockade therapy (ICB) was predicted using the TIDE score (http://tide.dfci.harvard.edu/login/) and immunophenoscore (IPS) (TCIA, https://tcia.at/patients).

### 2.13 Statistical analysis

Survival analysis was performed using the R survival package, and the survival rate of each group was evaluated using the log-rank test. Student T test and Wilcoxon test were used to compare data between groups. The Kaplan–Meier method was used to generate survival curves. The chi-square test was used to analyse the association of CRIRS subgroups and clinicopathological parameters. Pearson and Spearman methods were used for correlation analysis. All statistical analyses were performed using the R software. In the analysis of differences between cancerous and paraneoplastic tissues in PRAD, the screening condition was FDR < 0.05 and |log2 FC| > 1. A *P*-value of < 0.05 indicated significant differences in other analyses.

## 3 Results

### 3.1 Consensus clustering of patients with PRAD based on cuproptosis-related genes

**Figure 2:**
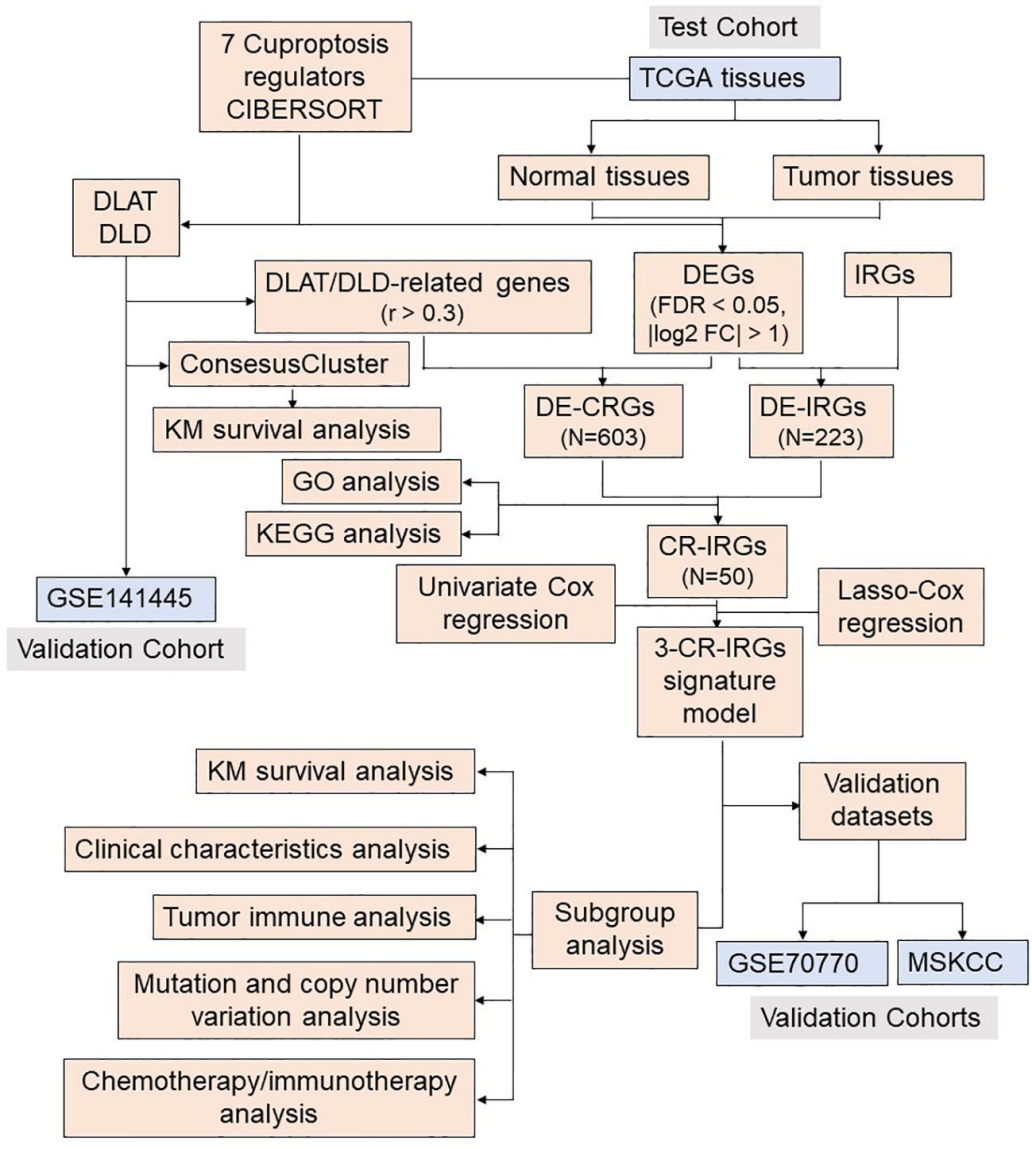
Flow chart of the analysis process.

**Figure 3:**
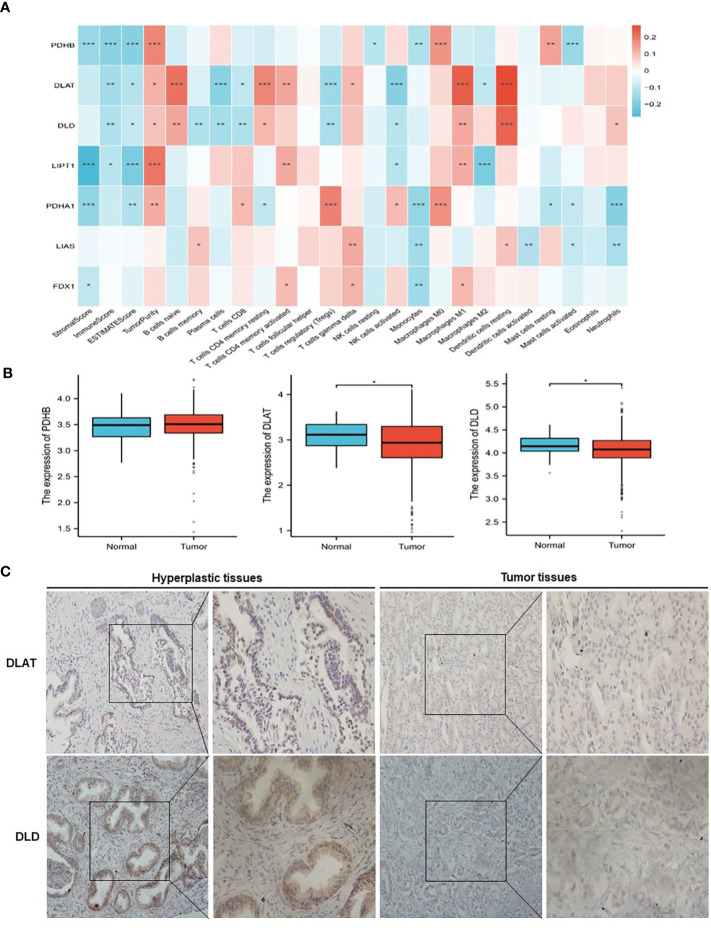
Classification of patients with PRAD in TCGA cohort according to the expression of DLAT and DLD. **(A)** Association of cuproptosis-related genes with the results of CIBERSORT. **(B)** Comparison of the expression of PDHB, DLAT and DLD between normal and PRAD tissues. **(C)** The protein levels of DLAT and DLD in prostate hyperplasia and prostate cancer clinical tissues were examined by immunohistochemistry. ^*^ *P* < 0.05, ^**^ *P* < 0.01, ^***^ *P* < 0.001.

**Figure 4:**
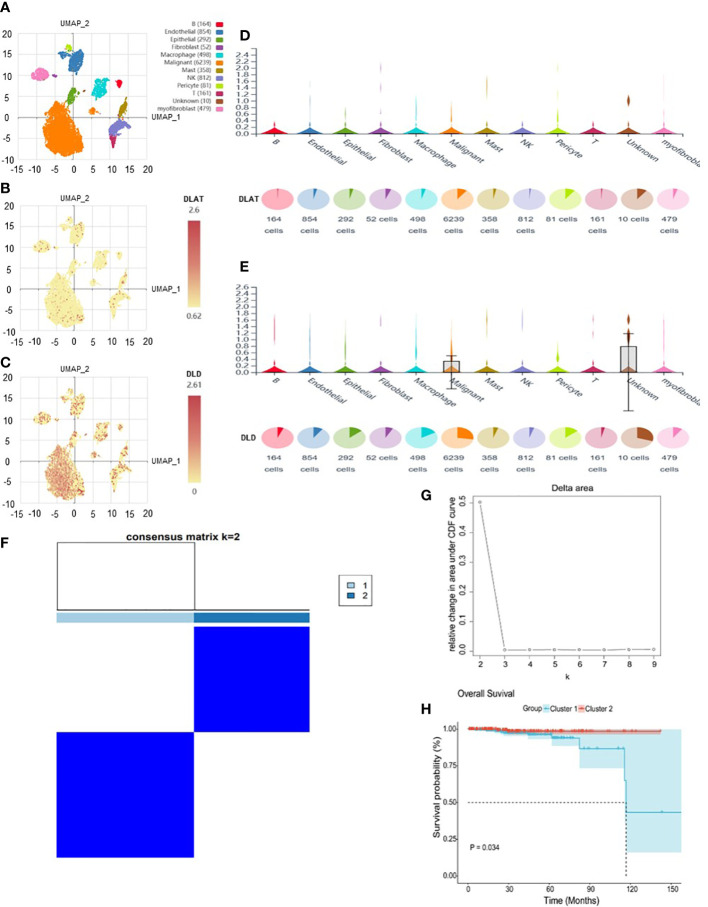
The expression of DLAT and DLD in immune cells in the GSE141445 dataset. **(A)** UMAP diagram of 13 samples. **(B, C)** UMAP distribution diagram showed the relative expression of DLAT and DLD in each cell. **(D, E)** Violin diagram showed the relative expression of DLAT and DLD in 8 types of cells. **(F, G)** Consensus matrix heat map defining two clusters (k = 2) and their correlation area. **(H)** Kaplan–Meier curves of overall survival in the two clusters.

The analysis flow chart of this study is shown in **Figure 2**. After excluding primary tumor samples without sufficient survival information, 499 samples were selected for follow-up analysis. To assess whether the expression of cuproptosis regulators affects the immune status of patients with PRAD, the expression of seven cuproptosis regulators, including FDX1, LIAS, LIPT1, DLD, DLAT, PDHA1 and PDHB, was compared among patients, and the immune cell infiltration levels of patients were calculated using the CIBERSORT algorithm. The results were ordered by absolute value of correlation with the ImmuneScore, and the expression of the seven cuproptosis regulators was significantly correlated with the infiltration of immune cells (**Figure 3A**). The expressions of the three highest correlated regulators with ImmuneScore, PDHB, DLAT and DLD, were compared among 550 samples. PDHB expression was not significantly different in cancerous and paracancerous tissues, and DLAT and DLD were significantly downregulated in cancerous tissues (**Figure 3B**). To further assess the expression of DLAT and DLD in prostate cancer tissues, we conducted IHC assays. Consistent with the aforementioned findings, our results indicated that DLAT and DLD expression was higher in benign prostatic hyperplasia tissues compared to prostate cancer tissues (**Figure 3C**). Next, the scRNA data were analyzed using the IMMUcan database to explore the expression of DLAT and DLD in the immune microenvironment of prostate cancer. **Figure 4A** shows the results of annotating prostate cancer cell types at the immune level. DLAT and DLD are expressed in both tumor cells and different types of stromal and immune cell subsets (**Figures 4B, C**). In stromal cell subpopulations, DLAT expression was mainly in fibroblasts, pericytes and myofibroblasts (**Figure 4D**), whereas DLD was mainly expressed in mast cells, NK cells and macrophages (**Figure 4E**). Subsequently, we selected DLAT and DLD to construct a risk profile and consensus clustering was performed to obtain two cuproptosis-associated clusters (**Figures 4F, G**). The survival of patients in the two clusters was analysed based on Kaplan–Meier curves. As shown in **Figure 4H**, patients in Cluster 2 had significantly better OS than patients in Cluster 1 (*P* = 0.034).

### 3.2 Identification and annotation cuproptosis- related and immune- related PRAD DEGs

**Figure 5:**
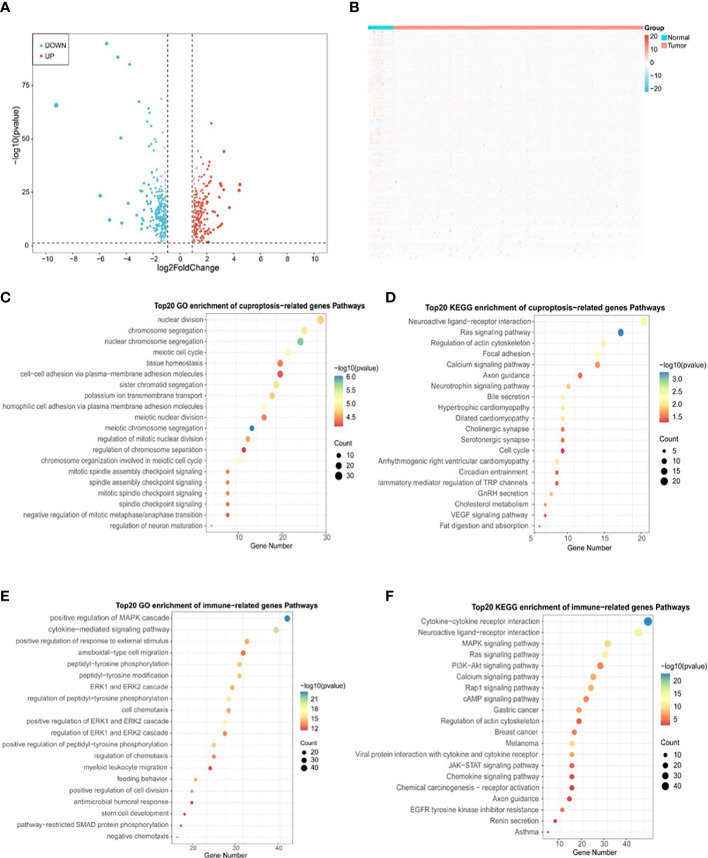
Identification of DLAT and DLD-related immune genes in TCGA-PRAD cohort. **(A)** Volcano plot of cuproptosis-related DEGs between normal and tumor tissues in TCGA-PRAD cohort. **(B)** Heatmap plot of immune-related DEGs between normal and tumor tissues in TCGA-PRAD cohort. **(C)** Top 20 terms for GO analysis of cuproptosis genes DLAT and DLD-related DEGs. **(D)** Top 20 pathways for KEGG analysis of cuproptosis genes DLAT and DLD-related DEGs. **(E)** Top 20 terms for GO analysis of immune-related DEGs. **(F)** Top 20 pathways for KEGG analysis of immune-related DEGs.

To determine the correlation between cuproptosis subtypes and immune function, 2483 IRGs were obtained from the ImmPort database. The ‘DESeq2’ package was used to identify differentially expressed genes (DEGs) in cancerous and paraneoplastic tissues (FDR < 0.05, |log2 FC| > 1). Further investigation of the relationship between PRAD DEGs and cuproptosis-related genes by Pearson correlation analysis showed 603 cuproptosis-related DEGs (CR-DEGs) (**Figure 5A**) (**Supplementary Table S2**). As shown in **Figure 5B**, 223 immune-related DEGs were screened in PRAD (IR-DEGs) (**Supplementary Table S3**). By taking the intersection of CR-DEGs and DE-IRGs, we identified 50 cuproptosis- related immune-related DEGs and were therefore referred to as CR-IRGs (**Supplementary Table S4**). Analysis of the GO and KEGG pathways of CR-DEGs and IR-DEGs showed intriguing results. Some of the pathways most enriched by CR-DEGs are overlapping with pathways associated with the most enriched by IR-DEGs, including Ras signaling pathway, Neuroactive ligand-receptor interaction, Regulation of actin cytoskeleton, Calcium signaling pathway and Axon guidance (**Figures 5C–F**), suggesting that the different cuproptosis states affecting PRAD prognosis may be associated with activation of immune pathways.

### 3.3 Construction of a prognostic model based on cuproptosis-related immune-related genes in TCGA-PRAD cohort

**Figure 6:**
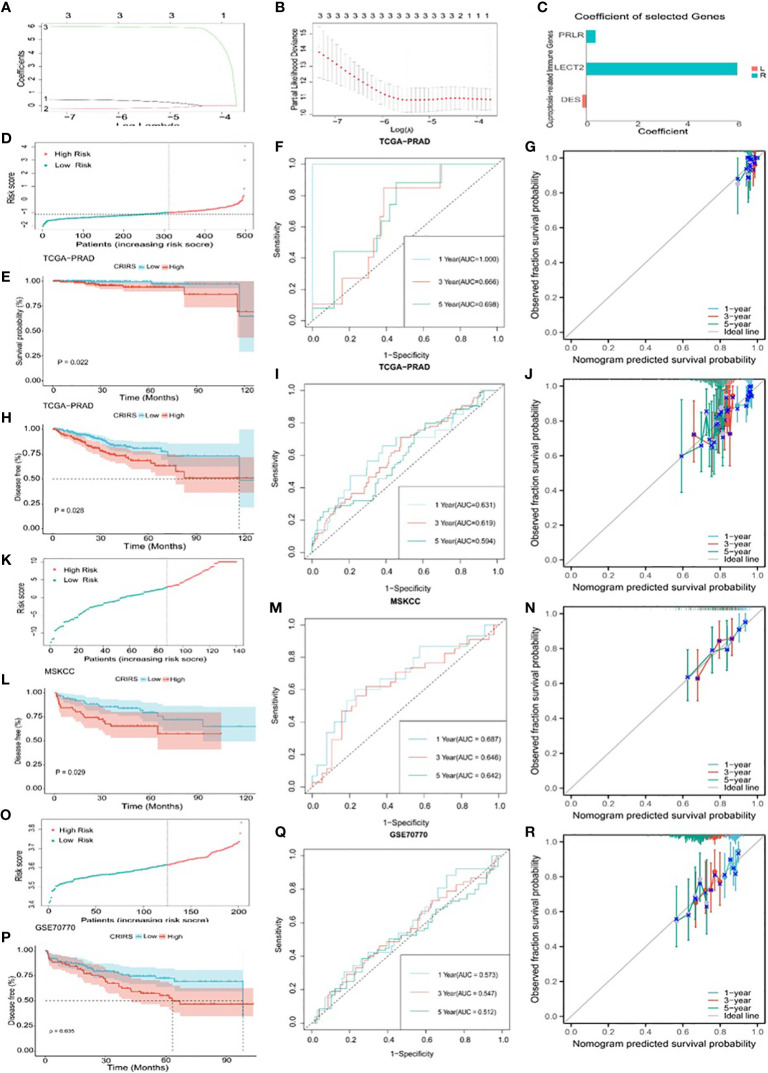
Construction and validation of a cuproptosis-related-IRG-based prognostic signature in TCGA-PRAD cohort. **(A, B)** DE-IRGs screened using a LASSO–Cox regression model. **(C)** Coefficients of three selected genes PRLR, LECT2, DES. **(D-J)** Construction of TCGA-PRAD training cohort. **(D)** Distribution and cut-off values of CRIRSs of TCGA training cohort. **(E)** OS of two CRIRS groups of TCGA-PRAD cohort. **(F)** ROC curves demonstrating the prognostic value of the CRIRS model in predicting 1-, 3- and 5-year OS in TCGA. **(G)** Calibration curves for CRIRS model of TCGA-PRAD cohort. y-axis: actual OS; x-axis: nomogram-predicted OS. **(H)** DFS of two CRIRS groups of TCGA-PRAD cohort. **(I)** ROC curves demonstrating the prognostic value of the CRIRS model in predicting 1-, 3- and 5-year DFS in TCGA. **(J)** Calibration curves for CRIRS model of TCGA-PRAD cohort. y-axis: actual DFS; x-axis: nomogram-predicted DFS. **(K-N)** Construction of MSKCC validation cohort. **(K)** Distribution and cut-off values of CRIRSs of MSKCC validation cohort. **(L)** DFS of two CRIRS groups of MSKCC cohort. **(M)** ROC curves demonstrating the prognostic value of the CRIRS model in predicting 1-, 3- and 5-year DFS in MSKCC. **(N)** Calibration curves for CRIRS model of MSKCC cohort. y-axis: actual DFS; x-axis: nomogram-predicted DFS. **(O-R)** Construction of GSE70770 validation cohort. **(O)** Distribution and cut-off values of CRIRSs of GSE70770 validation cohort. **(P)** DFS of two CRIRS groups of GSE70770 cohort. **(Q)** ROC curves demonstrating the prognostic value of the CRIRS model in predicting 1-, 3- and 5-year DFS in GSE70770. **(R)** Calibration curves for CRIRS model of GSE70770 cohort. y-axis: actual DFS; x-axis: nomogram-predicted DFS.

Based on the expression profiles of the 50 CR-IRGs, 3 significant CR-IRGs were initially screened *via* univariable Cox regression analysis (**Supplementary Table S5**). Subsequently, a prognostic model based on these genes was established *via* LASSO–Cox regression analysis (**Figures 6A–C**). Of the 499 patients, 311 patients (about 62%) were included in the high-risk group and 188 patients (about 38%) were included in the low-risk group (**Figure 6D**). Consistently, Kaplan–Meier curves showed that OS (*P* = 0.022) and DFS (*P* = 0.0028) were significantly worse in the high-CRIRS group than in the low-CRIRS group (**Figures 6E, H**). The OS and DFS predictive performance of the CRIRSs was assessed based on time-dependent ROC curves, and the area under the curve (AUC) values at 1, 3 and 5 years were 1.000, 0.666 and 0.698, and 0.631, 0.619 and 0.594, respectively (**Figures 6F, I**). The calibration curve shows that CRIRSs may accurately estimate the OS and DFS (**Figures 6G, J**). CRIRSs are calculated in the MSKCC and GSE70770 cohorts and validated by taking the same grouping approach as the TCGA-PRAD cohort (**Figures 6K, O**). Patients with lower CRIRSs had longer DFS in both MSKCC (*P* = 0.029) and GSE70770 (*P* = 0.035) cohorts (**Figures 6L, P**). Therefore, CRIRS was identified as a strong predictor of DFS, with AUC values of 0.687, 0.646 and 0.642 in MSKCC cohort and 0.573, 0.547 and 0.512 in the GSE70770 cohort at 1, 3 and 5 years, respectively (**Figures 6M, Q**). The calibration curves further validate the accurate predictive performance of CRIRSs for DFS (**Figures 6N, R**). These results illustrate the strong efficacy of the CRIRS model to predict the prognosis of prostate cancer.

### 3.4 Validation of the independent prognostic value of the 3-immune-gene signature

**Figure 7:**
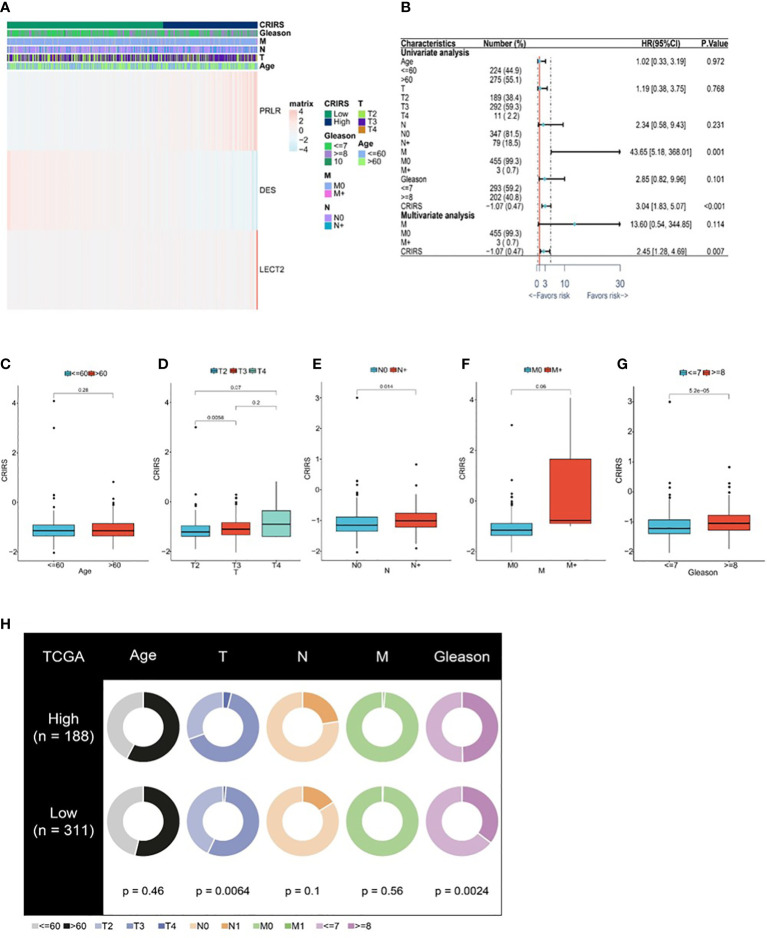
Correlation between CRIRS model and clinical characteristics based on TCGA-PRAD cohort. **(A)** Differences in clinicopathological features and expression levels of PRLR, LECT2 and DES between the low- and high-CRIRS groups. **(B)** Results of univariable and multivariable Cox regression analyses for predicting OS. Differences in CRIRS levels by age **(C)**, T stage **(D)**, N stage **(E)**, M stage **(F)**, and Gleason score **(G)** grouping. **(H)** Clinical characteristics of the high- and low-CRIRS groups.

**Figure 7A** illustrates the expression of PRLR and LECT2 was higher and that of DES was lower in the high-CRIRS group. Univariable and multivariable Cox regression analyses based on age, TNM stage, Gleason scores and CRIRSs revealed that the CRIRS was an independent prognostic factor for OS (**Figure 7B**). Additionally, To investigate whether CRIRS model correlated with the clinical characteristics of PRAD, we performed the Wilcoxon test and found that the high-CRIRS group had a later T stage (*P* = 0.0058) (**Figure 7D**), N stage (*P* = 0.014) (**Figure 7E**) and higher Gleason scores (*P* = 5.2e-05) (**Figure 7G**). However, age (**Figure 7C**) and M stage (**Figure 7F**) did not significantly differ between the two groups. The pieTable further demonstrates the significant correlation of CRIRSs with T stage (*P* = 0.0064) and Gleason scores (*P* = 0.0024) (**Figure 7H**). Additionally, we obtained 32 prostate cancer tissue samples to conduct correlation analysis between CRIRS and clinical parameters. The mRNA levels of PRLR, DES, and LECT2 were determined by qRT-PCR, while CRIRS was calculated using a specific formula. Results showed that in prostate cancer patients, CRIRS was positively correlated with their T stage (*P* = 0.033) and Gleason score (*P* = 0.025). However, no significant correlation was found between CRIRS and patients’ age and clinical stage (*P* > 0.05) (**Table 1**).

**Table 1:** Association of CRIRS with clinicopathological parameters in prostate cancer patients.

Characteristics	CRIRS (Low)(%)	CRIRS (High) (%)	*P*
n	20	12	
Age			0.399
≤ 60	3(15.0)	3(25.0)	
> 60	17(85.0)	9(75.0)	
T stage			**0.033**
T2	13(65.0)	3(25.0)	
T3	7(35.0)	9(75.0)	
Stage			0.227
II	9(45.0)	5(41.7)	
III-V	11(55.0)	7(58.3)	
Gleason Score			**0.025**
≤ 7	15(75.0)	4(33.3)	
≥ 8	5(25.0)	8(66.7)	

Bold values means P < 0.05.

### 3.5 Metabolic characteristics of patients classified based on CRIRSs

**Figure 8:**
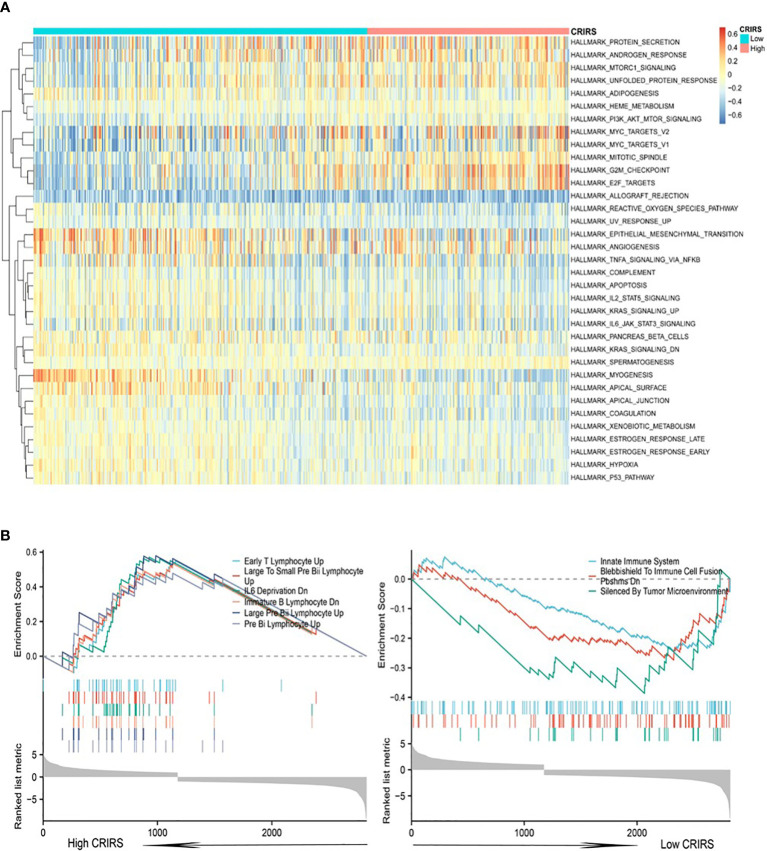
Enrichment analysis in the two CRIRS groups. **(A)** Analysis of multiple HALLMARK pathways *via* GSVA in the two CRIRS groups. **(B)** Immune-related pathways for GSEA enrichment analysis in two CRIRS groups.

Cuproptosis is associated with multiple cancer pathways (46). HALLMARK enrichment analysis showed that pathways related to tumor growth and invasion, such as mTORC1 signaling (47), PI3K/Akt/mTOR signaling (47), G2M checkpoint and Myc signaling (48) were significantly enriched in the high-CRIRS group (**Figure 8A**). Additionally, various immune activities, including complement, IL2/STAT5 signaling and IL6/Jak/STAT3 signaling, as well as metabolic pathways, such as spermatogenesis, myogenesis, and xenobiotic metabolism, were significantly enriched in the low-CRIRS group (**Figure 8A**). These findings explain, to some extent, the better prognosis of the low group. Subsequently, to further validate the function of the CRIRS model in terms of immunity, we performed GSEA pathway enrichment analysis and found six immune-related gene sets enriched in the high-CRIRS group, including Early T Lymphocyte Up, Large To Small Pre Bii Lymphocyte Up, IL6 Deprivation Dn, Immunature B Lymphocyte Dn and Pre Bii Lymphocyte Up. Three other immune-related pathways Innate Immune System, Blebbishield To Immune Cell Fusion Pbshms Dn and Silenced By Tumor Microenvironment were enriched in the low-CRIRS group (**Figure 8B**). Due to the complexity of enrichment of immune-related gene sets between the two CRIRS groups, we need further in-depth assessment of the immune status of the CRIRS model.

### 3.6 Correlation Between CRIRSs and the Tumor Microenvironment of PRAD

**Figure 9:**
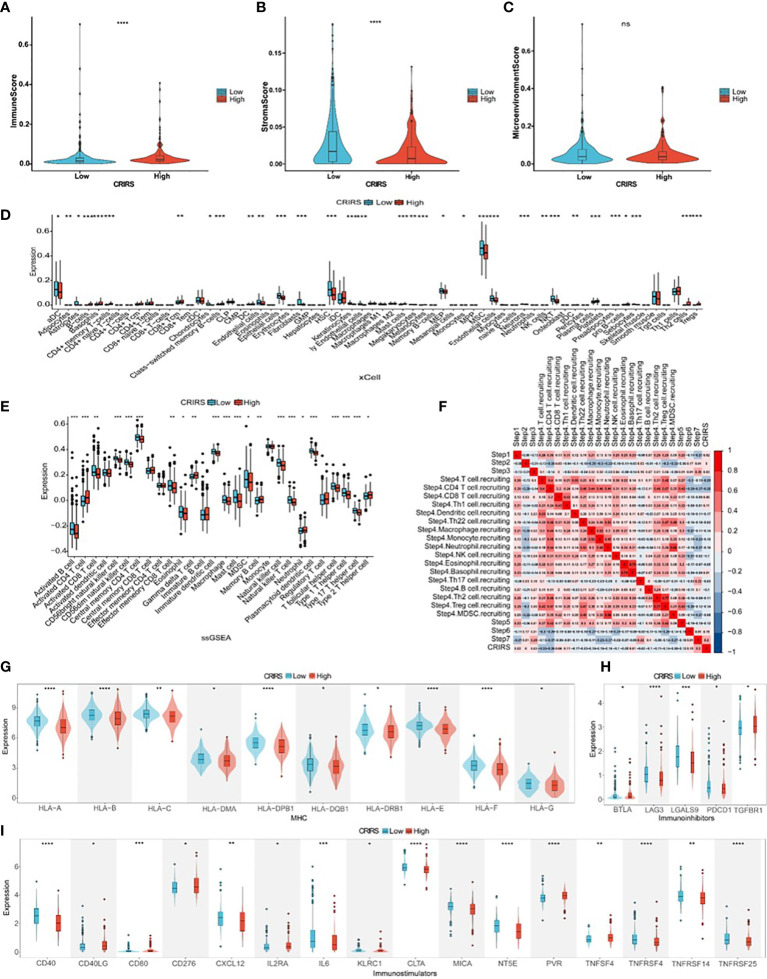
Comparison of immune activity in the two CRIRS groups. **(A-C)** Immune, stromal and microenvironment scores in the two CRIRS subtypes. **(D)** Different infiltration levels of 64 immune and stromal cells in the two CRIRS groups analysed using the xCell algorithm. **(E)** ssGSEA showed differences in the infiltration of immune cells between the two CRIRS groups. **(F)** Heatmap demonstrating correlation between seven key steps in the tumor immune cycle and CRIRSs. Differential expression of different types of immunomodulatory molecules MHC **(G)**, immunoinhibitors **(H)** and immunostimulators **(I)** in the two CRIRS groups. ^*^ *P* < 0.05, ^**^ *P* < 0.01, ^***^ *P* < 0.001, ^****^ *P* < 0.0001.

Several studies have shown that patients with higher immune scores and lower stromal scores have a better prognosis (49, 50). However, the low-CRIRS group with a better prognosis had higher stromal scores and lower immune scores, and no significant differences in immune microenvironment scores were observed between the low and high CRIRS groups in our study (**Figures 9A–C**). It has been showed that the density of infiltration of different immune cells in the center and invasive margins of tumors has different predictive significance for tumor prognosis and outcome due to the different immune structures of different tumors (51). This was also demonstrated in a study by Sun et al., kidney renal clear cell carcinoma patients who had a worse prognosis had higher immune scores and stromal scores (52). The relationship between CRIRSs and 64 types of adaptive and congenital immune cells, haematopoietic progenitor cells, epithelial cells and extracellular stromal cells was examined using the xCell algorithm. The proportion of multiple cell types was significantly different between the high- and low-CRIRS groups (**Figure 9D**). The proportion of multiple stromal cells including adipocytes, fibroblasts, lymphatic (ly) endothelial cells, and microvascular (mv) endothelial cells was high in the low-CRIRS group, whereas that of stem cells, such as hematopoietic stem cells (HSCs), megakaryocytes and megakaryocyte-erythroid progenitors (MEPs), and lymphoids NKT cells were also in higher proportions in the low-CRIRS group. Additionally, the proportion of a variety of lymphoids, such as B cells, CD4+ memory T cells, CD8+ Tcm, Th2 cells and Tregs, and some myeloids including Basophils and Mast cells, were highly represented in the high-CRIRS group. The ssGSEA analysis further demonstrates the infiltration of immune cells in two CRIRS groups. As shown in **Figure 9E**, activated B cells, activated CD8 T cells, CD56bright natural killer cells, CD56dim natural killer cells and natural killer cells was high in the low-CRIRS group, whereas that of activated CD4 T cells, memory B cells, neutrophils, regulatory T cells and type 2 T helper cells was high in the high-CRIRS group. The activity status of the seven-step tumor–immunity cycle of patients with PRAD was determined using the Tracking Tumor Immunophenotype (TIP) (http://biocc.hrbmu.edu.cn/TIP/) and visualised on a thermogram. Consistent with the above results, CRIRSs were negatively correlated with multiple step tumor–immunity cycle, especially in Step 4 (trafficking of immune cells to tumors) (**Figure 9F**). All three types of immune checkpoints, major histocompatibility complex (MHC), immunoinhibitors and immunostimulators, were highly expressed in the low-CRIRS group, especially HLA-A, HLA-B, LAG3, LGALS9, CD40 and CTLA (**Figures 9G–I**). These results reveal the reasons for the better prognosis in the low-CRIRS group.

### 3.7 Mutation landscape of patients classified based on CRIRSs

**Figure 10:**
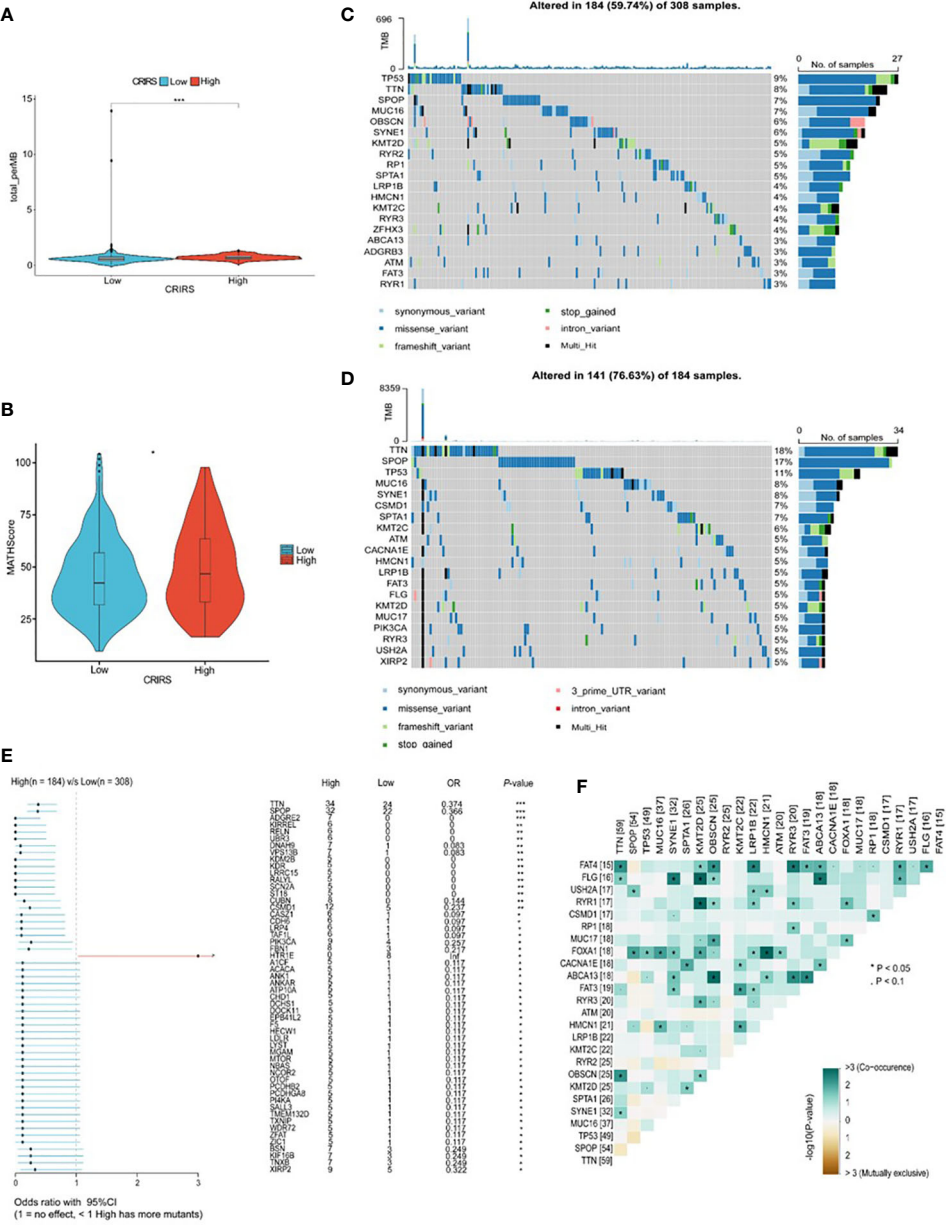
Genetic characteristics in the two CRIRS groups. **(A)** The distribution of TMB scores in the two CRIRS groups. **(B)** The distribution of MATH scores in the two CRIRS groups. **(C, D)** Waterfall plot of mutations in the top 20 genes in the low-CRIRS group top and high-CRIRS group bottom. **(E)** Forest plots demonstrating the frequency of 54 mutations that differed significantly between the two CRIRS groups. Higher mutation frequencies were found in the high-CRIRS group. **(F)** Heatmap demonstrating the commonality of mutations in the top 25 genes in PRAD. ^*^ *P* < 0.05, ***P < 0.001.

**Figure 11:**
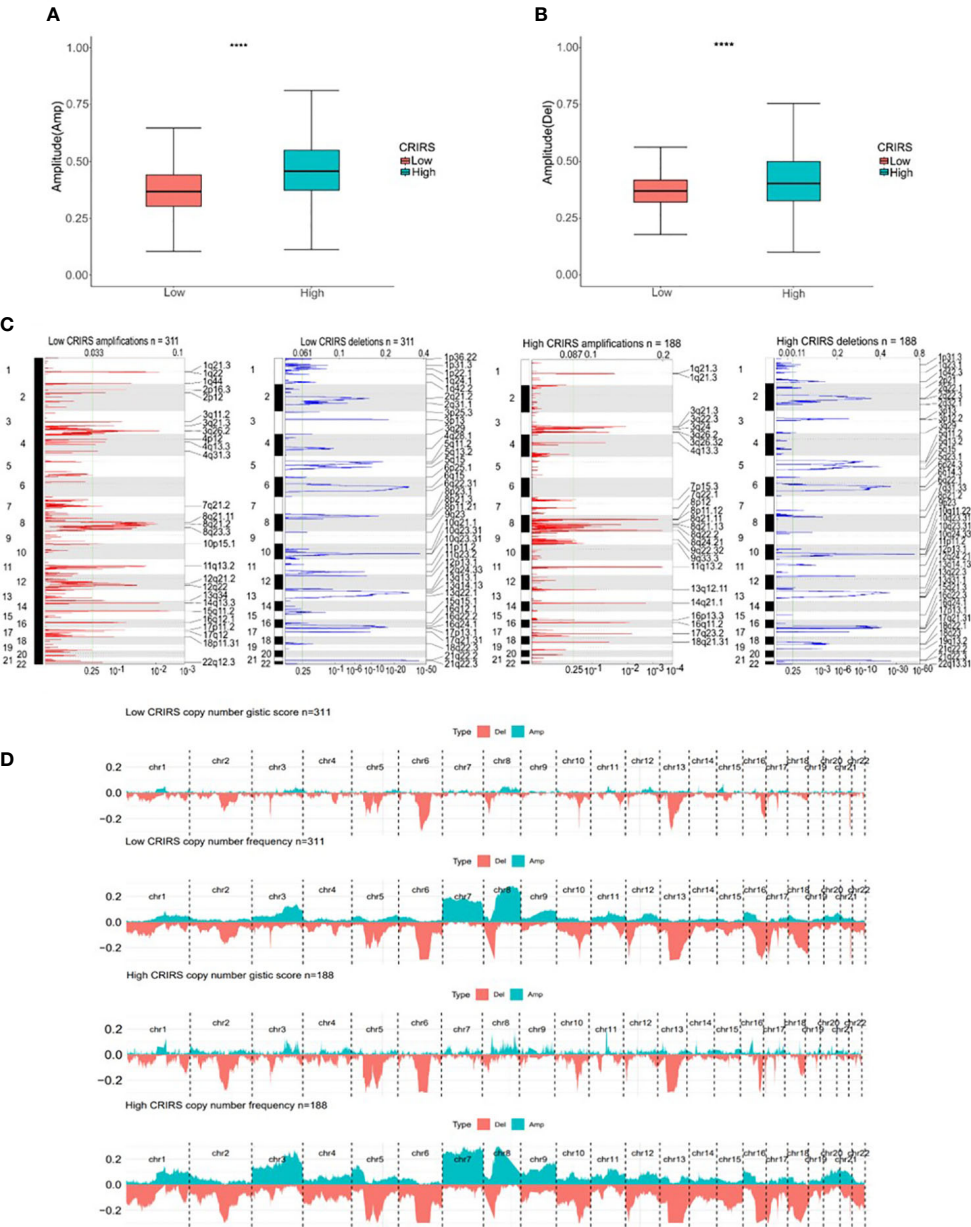
Genomic mutation profiles in the two CRIRS groups. **(A, B)** Box plot demonstrating the amplitudes of all chromosome amplifications/deletions in the two CRIRS groups. **(C)** Focal amplification/deletion of different chromosomal regions in the two CRIRS groups. **(D)** CNVs in the two CRIRS groups, including the logistic scores and mutation frequencies corresponding to different CNVs. ****P < 0.0001.

TMB and CNV in tumor patients correlate with prognosis (53). The mutation profile of patients stratified based on CRIRSs was examined. Higher TMB and MATH scores were observed in the high-CRIRS group (**Figures 10A, B**). The mutation profiles of patients were different between the two groups. As shown in **Figures 10C, D**, the top 5 genes with the highest mutation frequency were tumor protein P53 (TP53; 9%); titin (TTN; 8%); speckle-type BTB/POZ protein (SPOP; 7%); mucin 16, cell surface associated (MUC16; 7%) and titin-interacting RhoGEF (OBSCN; 6%) in the low-CRIRS group and TTN (18%); SPOP (17%); TP53 (11%); MUC16 (8%) and spectrin repeat containing nuclear envelope protein 1 (SYNE1; 8%) in the high-CRIRS group. TTN mutations were found in 34 patients in the high-CRIRS group and 24 patients in the low-CRIRS group (odds ratio [OR] = 0.374, *P* < 0.01, **Figure 10E**). The mutation frequency of HTR1E was high in the low-CRIRS group (*P* < 0.05), whereas mutation frequencies in 53 genes including SPOP, ADGRE2 and KIRREL were higher in the high-CRIRS group (*P <* 0.05, **Figure 10E**). Co-mutation relationships were observed between multiple genes and the five genes with the highest mutation frequencies: TTN mutations were related to FAT4, FLG, OBSCN and SYNE1 mutations; SPOP mutations were related to USH2A and FOXA1 mutations, TP53 mutations were related to FOXA1 mutations; MUC16 mutations were related to FOXA1 and HMCN1 mutations; and SYNE1 mutations were related to FLG, FOXA1, ABCA13 and FAT3 (**Figure 10F**). Given that CNVs may lead to chromosomal alterations, we further investigated the relationship between CRIRSs and CNVs. The frequency of CNV amplification and deletion was significantly high in the high-CRIRS group (**Figures 11A–C**). **Figure 11D** shows the topography of CNVs in the high- and low-risk groups. More genes had CNV amplification and deletion in the high-CRIRS group than in the low-CRIRS group.

### 3.8 Predicting the sensitivity of patients to antitumor therapy

**Figure 12:**
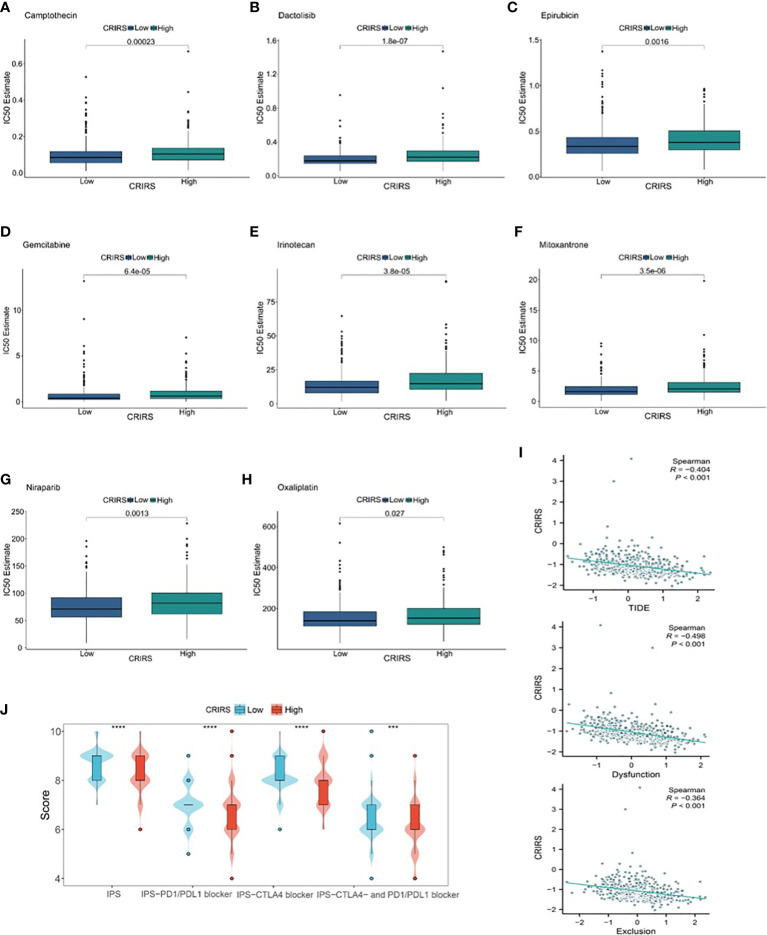
Assessment of chemotherapy and immunotherapy responses in the two CRIRS groups. **(A–H)** The response of patients to eight common chemotherapeutic drugs in the high- and low-CRIRS groups. **(I–J)** Immunotherapy response prediction in the two CRIRS groups. ***P < 0.001, ****P < 0.0001.

The IC50 values of several chemotherapeutic agents commonly used in the treatment of PRAD were evaluated to predict the response of patients with different CRIRSs to antitumor therapy. The IC50 values of Camptothecin (*P* = 0.00623) (**Figure 12A**), Dactolisib (*P* = 1.8e-07) (**Figure 12B**), Epirubicin (*P* = 0.0016) (**Figure 12C**), Gemcitabine (*P* = 6.4e-05) (**Figure 12D**), Irinotecan (*P* = 3.8e-05) (**Figure 12E**), Mitoxantrone (*P* = 3.5e-06) (**Figure 12F**), Niraparib (*P* = 0.0013) (**Figure 12G**) and Oxaliplatin (*P* = 0.027) (**Figure 12H**) were significantly higher in the high-CRIRS group than in the low-CRIRS group. In addition, TIDE analysis showed that CRIRS was significantly and negatively correlated with TIDE, Dysfunction and Exclusion scores (**Figure 12I**). However, IPS scores were higher in the low-CRIRS group, indicating a better response to immunotherapy in the low-CRIRS group (**Figure 12J**).

## 4 Discussion

Unbalanced copper homeostasis can affect tumor growth and induce tumor cell death (54). Copper also plays an integral role in tumor immunity and antitumor therapy (55, 56). Cuproptosis plays a complex regulatory role in the TME of various cancers such as endometrial and colorectal cancers. However, its role in the development of TME and its potential therapeutic value in PRAD remain unclear. Multiple risk models based on cuproptosis-associated genes can accurately predict prognosis and assess the tumor microenvironment (57, 58). Zhu et al. reported that the three cuproptosis patterns they constructed in colorectal cancer were consistent with the results of immune infiltration characteristics (59).

In this study, we proposed a cuproptosis-related immune scoring system to assess individual immune profiles. Immune regulation was analyzed based on transcriptional changes and the expression of cuproptosis-related genes in TCGA-PRAD cohort. The cuproptosis genes DLAT and DLD were found to be closely associated with PRAD. An unsupervised clustering approach was used to divide TCGA-PRAD cohort into two differentially characterized cuproptosis clusters based on the expression of DLAT and DLD. Prognosis was significantly different between the two groups. Based on cuproptosis-related IRGs, three genes associated with different clinical outcomes, immune activity and immune function were identified, namely, PRLR, DES and LECT2. These three genes play an important role in tumor immunity. It has been reported that PRLR might affect the prognosis of breast cancer by inhibiting the expression of immune checkpoints (60). Liu et al. demonstrated that TP53-associated immune prognostic model (TIPM) including PRLR predicts overall survival and treatment response in pancreatic cancer (61). Absence of Reed-Sternberg cell DES and cytokeratin expression in Hodgkin’s disease with Ki-1 antigen expression may be associated with dysregulation of the immune system and the observed immunological abnormalities (62). Pouyanfard et al. demonstrated that treatment of liver fibrosis with a population of human iPSC-derived M2 subtype macrophages in an immunodeficient Rag2 γc mouse model significantly reduced the expression of fibrotic genes, including DES (63). LECT2 deficiency fosters the accumulation of pejorative inflammatory monocytes harboring immunosuppressive properties and strong tumor-promoting potential in hepatocellular carcinoma (64). Qin et al. reported that LECT2 expression was low in hepatocellular carcinoma and negatively correlated with the infiltration of immune cells such as B cells, neutrophils and monocytes and positively correlated with naïve CD8 T cells, endothelial cells and hematopoietic stem cells (65).

The CRIRS system was established *via* LOSSO–Cox regression analysis. High CRIRSs were associated with shorter OS and DFS. GSEA revealed that multiple cancer-related pathways were significantly enriched in the high-CRIRS group, suggesting that the three cuproptosis-associated IRGs are involved in tumor development. CRIRSs were significantly correlated with the clinicopathological features of PRAD, such as T stage and Gleason scores. After controlling for confounding factors, CRIRS was identified as an independent predictor of survival outcomes in PRAD. ROC curves and Calibration curves demonstrated that CRIRSs had good accuracy in predicting OS and DFS at 1, 3 and 5 years. Therefore, CRIRSs may serve as an effective tool to predict the prognosis of PRAD. Significant differences were observed in the frequency of gene mutations between the high- and low-CRIRS groups. Multiple genes had higher mutation frequencies in the high-CRIRS group. CNVs are one of the most important somatic aberrations in cancer, which contribute to the pathogenesis of many disease phenotypes. In this study, the frequency of CNV amplification and deletion was high in the high-CRIRS group.

The immune response plays a dominant role in tumorigenesis and can often serve as the target of tumor therapy. Immune and stromal cells are major components of TME (66). Our study found that the CIBERSORT algorithm showed zero abundance of T cell CD4 naive infiltration, probably because CIBERSORT calculated the relative proportions of immune cell subpopulations in tumor tissues instead of the actual values (67). Immune cell infiltration is associated with the prognosis of PRAD, and high infiltration levels of CD8^+^ T cells and NK cells may indicate a good prognosis, which is consistent with the results of this study (68–70). Therefore, cuproptosis may be involved in regulating TME, especially CD8^+^ T cells and NK cells, thereby promoting tumor growth and progression. Previous studies have reported that reactivation of CD8+ T cells can indicate the efficacy of immunotherapy. Therefore, targeting cuproptosis-related IRGs may be an effective and novel therapeutic strategy for the treatment of PRAD.

Chemotherapy and androgen deprivation therapy may limit tumor progression and improve the prognosis of patients with PRAD (71, 72). At present, the decreasing sensitivity of PRAD to chemotherapy is a major concern worldwide (73). The ‘cold’ tumor characteristics of PRAD inhibit the development of immunotherapeutic strategies that can optimize treatment by driving T cells into the tumor and transforming the ‘cold’ TME into an immune ‘hot’ TME (74). In this study, patients in low-CRIRS groups were potentially sensitive to several therapeutic drugs, which may help to mitigate resistance mechanisms and improve clinical outcomes. To investigate whether CRIRSs can help to predict the efficiency of immunotherapy in PRAD, the correlation between CRIRSs and 31 immune checkpoint genes was examined. The vast majority of these genes were highly expressed in the low-CRIRS group. The TIDE algorithm and IPS scores were used to predict the ICB responses of patients with the low-CRIRS group with higher IPS predicted a better response to immunotherapy.

This study has some limitations. First, individual differences among patients with PRAD might have affected the cuproptosis-associated IRG-based prognostic signature, and more external and practical validation is required to determine whether the signature can be used in clinical practice. In addition, we have only limited knowledge of the signalling pathways related to the three cuproptosis-associated IRGs identified in this study, and the specific molecular mechanisms of these genes in PRAD and their relationship with TME and cuproptosis remain unknown. The role of these genes in PRAD should be examined *in vivo* and *in vitro* in future studies using the results of GSEA as a reference.

## Data availability statement

The datasets used in this study TCGA-PRAD (https://portal.gdc.cancer.gov/) were accessed online on September 30, 2022, MSKCC (https://www.cbioportal.org/) GSE70770 (https://www.ncbi.nlm.nih.gov/geo/query/acc.cgi?acc=GSE70770) and GSE141445 (https://www.ncbi.nlm.nih.gov/geo/query/acc.cgi) were accessed online on December 17, 2022.

## Author contributions

KY participated in the study conception, data analysis and visualization. RZ performed data collection and visualization. LL and ML participated in data analysis, SF and HY analyzed the data and prepared the manuscript. ZZ participated in data analysis and manuscript revision. DX contributed to study design and writing. All authors contributed to the article and approved the submitted version.
