# Supplementary material for: The signature of cuproptosis-related immune genes predicts the tumor microenvironment and prognosis of prostate adenocarcinoma
Source: Front Immunol. 2023 Aug 2;14:1181370. doi: 10.3389/fimmu.2023.1181370 (PMC10433769; doi:10.3389/fimmu.2023.1181370)
Supplement: Supplementary file 1 [file DataSheet_1.zip › Supplementary Tables/Supplementary Table S1.docx]

**Supplementary Table S1: Association of CRIRS with clinicopathological parameters in prostate cancer patients.**

| **Characteristics** | **TCGA** | **GSE70770** |
| --- | --- | --- |
| n | 499 | 293 |
| Age |  |  |
| ≤ 60 | 224 | 49 |
| > 60 | 275 | 244 |
| T stage |  |  |
| T1-T2 | 189 | 176 |
| T3-T4 | 303 | 25 |
| N Stage |  |  |
| N0 | 347 | 39 |
| N+ | 79 | 2 |
| Nx | / | 69 |
| Gleason Score |  |  |
| ≤ 7 | 293 | 180 |
| ≥ 8 | 202 | 33 |
